# Exercise training modifies xenometabolites in gut and circulation of lean and obese adults

**DOI:** 10.14814/phy2.15638

**Published:** 2023-03-22

**Authors:** Mikaela C. Kasperek, Lucy Mailing, Brian D. Piccolo, Becky Moody, Renny Lan, Xiaotian Gao, Diego Hernandez‐Saavedra, Jeffrey A. Woods, Sean H. Adams, Jacob M. Allen

**Affiliations:** ^1^ Division of Nutritional Sciences University of Illinois at Urbana‐Champaign Urbana Illinois USA; ^2^ Department of Kinesiology and Community Health University of Illinois at Urbana‐Champaign Urbana Illinois USA; ^3^ Arkansas Children's Nutrition Center Little Rock Arkansas USA; ^4^ Department of Pediatrics University of Arkansas for Medical Sciences Little Rock Arkansas USA; ^5^ Department of Surgery University of California, Davis School of Medicine Sacramento California USA; ^6^ Center for Alimentary and Metabolic Science University of California, Davis Sacramento California USA

**Keywords:** exercise, metabolites, microbiome, obesity, xenometabolism

## Abstract

Regular, moderate exercise modifies the gut microbiome and contributes to human metabolic and immune health. The microbiome may exert influence on host physiology through the microbial production and modification of metabolites (xenometabolites); however, this has not been extensively explored. We hypothesized that 6 weeks of supervised, aerobic exercise 3×/week (60%–75% heart rate reserve [HRR], 30–60 min) in previously sedentary, lean (*n* = 14) and obese (*n* = 10) adults would modify both the fecal and serum xenometabolome. Serum and fecal samples were collected pre‐ and post‐6 week intervention and analyzed by liquid chromatography/tandem mass spectrometry (LC–MS/MS). Linear mixed models (LMMs) identified multiple fecal and serum xenometabolites responsive to exercise training. Further cluster and pathway analysis revealed that the most prominent xenometabolic shifts occurred within aromatic amino acid (ArAA) metabolic pathways. Fecal and serum ArAA derivatives correlated with body composition (lean mass), markers of insulin sensitivity (insulin, HOMA‐IR) and cardiorespiratory fitness ($\dot{V}O_{2\max}$), both at baseline and in response to exercise training. Two serum aromatic microbial‐derived amino acid metabolites that were upregulated following the exercise intervention, indole‐3‐lactic acid (ILA: fold change: 1.2, FDR *p* < 0.05) and 4‐hydroxyphenyllactic acid (4‐HPLA: fold change: 1.3, FDR *p* < 0.05), share metabolic pathways within the microbiota and were associated with body composition and markers of insulin sensitivity at baseline and in response to training. These data provide evidence of physiologically relevant shifts in microbial metabolism that occur in response to exercise training, and reinforce the view that host metabolic health influences gut microbiota population and function. Future studies should consider the microbiome and xenometabolome when investigating the health benefits of exercise.

## Graphical abstract

Xenometabolomic responses to exercise were different between lean and obese adults, despite some shared adaptations. Microbial metabolites associated with body composition, markers of insulin sensitivity, and cardiorespiratory fitness, both at baseline and in response to exercise training. These data suggest microbial metabolites should be carefully considered as contributors to exercise adaptations and human health.

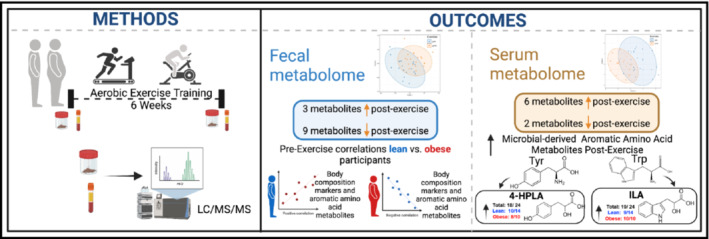

## 1 INTRODUCTION

The gut microbiota is a malleable ecosystem of bacteria and other microorganisms that are major contributors to human health. Gut microbes influence host physiology partially through the production and modification of a wide range of bioactive metabolites. In mammals, it has been estimated that ~10% of all circulating metabolites are microbially derived (Wikoff et al., 2009). These “non‐host” synthesized molecules are part of a broad class of “xenometabolites” (Campbell et al., 2014; Mercer et al., 2020) and include bioactive metabolites derived from the fermentation of carbohydrates, amino acids, bile acids, and lipids that modify host physiology. Because disturbances to gut microbiota metabolism are associated with a wide variety of human health conditions, it is imperative to understand how lifestyle factors—including exercise—can contribute to microbial metabolic output and signaling.

Participation in regular, moderate exercise reduces the incidence of metabolic and inflammatory disease, including gastrointestinal diseases such as inflammatory bowel disease and colorectal cancer (Bilski et al., 2018; Lambdin et al., 2018; Ng et al., 2007). Physical activity is known to impact gut microbiome composition and function (Allen et al., 1985; 2018; Anhe et al., 2022; Barton et al., 2017; Campbell et al., 2016; Matsumoto et al., 2008), which may potentially confer some of the health benefits of exercise. For instance, a recent cross‐sectional study has shown that both fecal microbiome richness and microbe‐derived metabolites such as butyrate were significantly associated with cardiorespiratory fitness (V̇O_2peak_) (Estaki et al., 2016). In addition, women with high aerobic capacity, when compared to age‐ and body mass‐matched women with low fitness, displayed a significant increase in plasma concentrations of a secondary bile acid, lithocholic acid, following an oral glucose tolerance test (OGTT) (Maurer, 1985). In contrast, post‐OGTT serum total conjugated bile acids were only transiently increased in more fit women whereas in less fit women levels rose and remained high (Maurer, 1985).

While longitudinal studies are relatively sparse in humans, exercise training appears to affect the microbiome function over time in previously sedentary individuals. For instance, our previous work showed that exercise training increases fecal short‐chain fatty acids (SCFAs) (Allen et al., 2018): microbe‐derived xenometabolites that have anti‐inflammatory, satiety, and insulin‐sensitizing effects (Cox et al., 2009; Larraufie et al., 2018; Zarrinpar et al., 2018). Additional evidence from fecal transfer treatments showed that exercise‐induced increases in SCFAs may contribute to improvements in insulin sensitivity in pre‐diabetic subjects (Liu et al., 2020). Furthermore, following a weight loss and fitness intervention in previously sedentary, insulin‐resistant women, a variety of plasma xenometabolite patterns were shifted despite the fact that participants were consuming the same foods leading up to the metabolomics studies (Campbell et al., 2014; Zhang, 2017). Because previous exercise training studies have primarily been limited to targeted pathway and metabolite analysis, controlled longitudinal human exercise studies focused on global xenometabolic responses are needed.

Obesity rates are growing worldwide and are associated with disturbances in gut microbiota metabolism (Wang et al., 2021). In mice, 6 weeks of high‐intensity exercise training has been shown to directly oppose many of the obesity‐related changes in the gut microbiota and microbial metabolism (Denou et al., 2016). In particular, regular exercise training increased the predicted genetic capacity related to glycan biosynthesis and metabolism, carbon fixation, and the TCA cycle in the fecal microbiota. While a defined microbial signature of obesity has not yet been identified in humans, there is evidence of altered metabolic output in the gut microbiome of obese humans (de Vadder & Mithieux, 2018). Similarly, the success in weight loss interventions, including calorie restriction and bariatric surgery, have been tightly linked to changes in gut microbially derived xenometabolites, including secondary bile acids and SCFAs (Heianza et al., 2018; Ocana‐Wilhelmi et al., 2021; Seyfried et al., 2021). It is thus vital to understand whether obesity‐associated metabolites derived from the microbiome are responsive to exercise training interventions in humans, since this might contribute to the known metabolic health benefits of exercise in this population. Our previous data revealed an orthogonal microbiome response to exercise training in humans that was dependent on obesity status (Allen et al., 2018). Most notably, lean participants exhibited a more substantial increase in SCFAs than obese participants in response to exercise training, suggesting that obesity may influence metabolic responses to exercise training. However, to date, no studies have determined the effect of obesity status on global xenometabolite responses to exercise training.

In the present study, we built upon our previous research to analyze how a 6 week, supervised aerobic exercise program modifies the fecal and serum metabolome of previously sedentary, lean and obese humans. Using untargeted metabolomic profiles, we sought to understand the changes in the global metabolome (including the xenometabolome) in the gut and circulation in response to exercise training.

## 2 METHODS

### 2.1 Participants

Serum and fecal biospecimens from a previously published study were used in this analysis (Allen et al., 2018). Participants (*n* = 24 total) were age 20 to 45 year, had a BMI <25 kg/m^2^ (lean; *n* = 15) or a BMI >30 kg/m^2^ (obese; *n* = 9), and were previously sedentary as defined as ≤30 min of moderate‐ or high‐intensity exercise per week and ≤10 aggregate Godin‐Shepard Leisure Time Physical Activity Questionnaire (GSLTQ) score (Table 1). Participant medical history and medication use were assessed by questionnaire. Subjects who qualified for the study were free of metabolic and gastrointestinal disease, not pregnant or lactating, not taking medications that would impact bowel function, and had not taken antibiotics for at least 3 months prior to beginning the study.

**TABLE 1:** Subject characteristics at baseline and in response to 6‐week exercise training

	Baseline		Post‐exercise intervention	
	Lean (*n* = 15); 7 female	Obese (*n* = 9); 7 female	Lean (*n* = 15); 7 female	Obese (*n* = 9); 7 female
Age (year)	23.64 ± 0.91	28.10 ± 2.72		
BMI (kg m^−2^)	21.56 ± 0.73	33.76 ± 1.98*	22.11 ± 0.91	35.15 ± 3.48*
Weight (kg)	67.34 ± 3.63	92.58 ± 6.99*	68.11 ± 3.90	91.31 ± 6.70*
Body fat %	23.84 ± 1.03	36.85 ± 1.71*	23.26 ± 0.96	35.69 ± 1.73*,**
Lean mass %	73.78 ± 1.02	60.96 ± 1.71*	74.33 ± 0.94	62.27 ± 1.74*
Bone density (g cm^−2^)	1.12 ± 0.02	1.18 ± 0.03	1.12 ± 0.02	1.19 ± 0.03**
Absolute $\dot{V}O_{2\max}$	2.62 ± 0.19	2.77 ± 0.25	3.07 ± 0.20	2.96 ± 0.19
Relative $\dot{V}O_{2\max}$	40.15 ± 1.33	30.49 ± 2.09**	45.28 ± 1.56	34.17 ± 2.29*,**
Glucose (mg dL^−2^)	88.78 ± 2.85	89.66 ± 3.19	80.13 ± 3.64	83.46 ± 6.31
Insulin (mIU mL^−2^)	9.90 ± 0.51	13.46 ± 1.48*	9.75 ± 0.57	12.12 ± 1.05
HOMA‐IR	2.16 ± 0.11	3.02 ± 0.42	1.94 ± 0.16	2.55 ± 0.35

*Note*: Data are mean ± SEM.

* Significant effect of BMI at *p* < 0.05

** Significant effect of exercise at *p* < 0.05.

### 2.2 Exercise training protocol

The exercise intervention consisted of supervised 30 to 60 min, moderate‐to‐vigorous intensity (60%–75% of HR reserve [HRR]) aerobic exercise sessions. Subjects exercised 3×/week for 6 weeks and chose from a cycle ergometer or treadmill during each session. Training sessions for the first two weeks were 30–45 min at 60% HRR and at week 3 were increased to 60 min at 60% HRR. During weeks 4 to 6 of training, there was an increase in intensity of 5% HRR per week, progressing up to 75% HRR for 60 min during week 6. All participants were 100% compliant in completing necessary requirements for the exercise portion of the study. The participants completed the study in two separate cohorts within a 6 month period in 2016. Study cohort was accounted for in all statistical modeling.

### 2.3 Screening and diet control

In consultation with a registered dietitian, participants designed a 3‐day food menu that consisted entirely of foods and drinks from a 7‐day dietary recall of detailed descriptions of the types and amounts of foods and beverages consumed. Participants were asked to follow this 3‐day menu before each fecal and blood collection to ensure lead‐in conditions were controlled and reflected typical dietary intake. In addition to the acute dietary control, subjects were instructed to maintain overall dietary patterns, including maintenance of alcoholic and caffeinated beverage consumption, and continuation of any dietary supplements that occurred before the study commenced.

### 2.4 Biospecimen collection

Fecal samples were collected at baseline and after 6 weeks of exercise training and were preceded by the individualized 3‐day diet control and were collected within 24 h of blood collection as previously described (Allen et al., 2018). Subjects were provided with fecal sample collection containers and were instructed to deliver samples to the laboratory within 30 min of defecation to ensure minimal degradation of volatile SCFAs. Once received, ~0.5 g of the sample was aliquoted for SCFA analysis and the rest was stored at −80°C until future analysis. Fasted (>/=10 h) blood samples were collected before 9:00 am at baseline and after the exercise intervention.

### 2.5 Metabolomics

Frozen serum and stool samples were shipped to the Arkansas Children's Nutrition Center (ACNC) overnight on dry ice and immediately stored at −80°C upon arrival. Full analytical details of the metabolomics methods have been previously published (Mercer et al., 2020; Piccolo et al., 2017) with slight modifications. Stool and serum samples were analyzed separately. A portion of stool sample for each participant was pulverized into powder using a SPEX CertiPrep 6750 freezer mill and then stored at −80°C until analysis. Powdered stool samples (~100 mg) were dried overnight under a slow nitrogen stream to remove water content and ~30 mg of the dried powder was homogenized in 500 μL 50% aqueous methanol using a Precellys 24 homogenizer (Bertin Corp.) with 2.8 mm ceramic beads at 5300 rpm for two 30‐s cycles. Samples were then extracted in 1 mL of ice‐cold acetonitrile. Serum samples (100 μL) were extracted directly in methanol (1:4). Quality control (QC) samples were prepared by pooling equal volumes (10 μL each) of all extracted samples. Samples and QC extracts were then evaporated to dryness using a nitrogen gas evaporator. Dried extracts were reconstituted in 5% aqueous methanol (150 μL for serum and 300 μL for fecal powder) containing internal standards [1 μg/mL Lorazepam, D6‐trans‐cinnamic acid, and D4 glycocholic acid (Sigma Aldrich, St. Louis, MO)]. Group pooled samples were made by mixing 10 μL of each reconstituted sample from both pre‐ and post‐exercise groups. Samples were placed in random sequence for analysis. A Dionex Ultimate 3000 UHPLC with a XSelect CSH C18 reversed phase column (2.1 × 100 mm, 2.5 μm; 49°C) was used for chromatography, followed by detection using a Q‐Exactive Hybrid Quadrupole‐Orbitrap mass spectrometer. Metabolites were eluted by use of the following step gradient at a flow rate of 0.4 mL/min: 0–2 min, 0%–1% solvent B; 2–6.5 min, 1%–20% solvent B; 6.5–11.5 min, 20%–95% solvent B; 11.5–13.5 min, 95%–99% solvent B; 13.5–16.5 min, 99%–1% solvent B; 16.5–20 min, 1% solvent B; 20–21 min, 1%–0% solvent B; 21–22 min, 0% solvent B (Solvent A is 0.1% by volume formic acid in water and solvent B is 0.1% by volume formic acid in acetonitrile). Injection volumes were set to 5 μL. All of the samples were analyzed by positive and negative electrospray ionization (ESI+/−) in full scan MS mode with the polarity switching. The QC sample was used to condition the system and was also injected between every 15 sample injections. Nitrogen as sheath, auxiliary, and sweep gas were set at 50, 13, and 3 U, respectively. Other conditions included the following: resolution, 70,000 full width at half maximum (FWHM); automatic gain control target, 3e6 ions; maximum injection time, 200 ms; scan range, 50–750 *m/z*; spray voltage, 3.50 kV; and capillary temperature, 320°C. ESI+/− data‐dependent MS–MS spectra were generated for QC pool samples as well as group pooled samples through the use of the following conditions: resolution, 17,500 FWHM; automatic gain control target, 1e5 ions; maximum injection time, 50 ms; isolation window, 4 Da; and NCE at 30. Data were acquired as full MS and data‐dependent MS^2^ using Xcalibur 4.0 software.

#### 2.5.1 Data processing and metabolite identification

The acquired data set, composed of full MS and data‐dependent MS–MS raw files, was processed using Compound Discoverer 3.0 (Thermo) to perform peak alignment, peak picking, compound grouping, and quantification for each metabolite. Details of data workflow have been previously published (Piccolo et al., 2017) with a slight modification. The software parameters for detecting unknown compounds were 5 ppm mass tolerance for detection, 30% intensity tolerance, 3 for the sensitivity and noise threshold, and 5e5 minimum peak intensity. Spectral information and retention times were matched to the library of authentic standards at the ACNC using MassList (accurate mass ± 5 ppm, RT ± 15 s). The ACNC authentic in‐house standard library (mzVault) contains 420 metabolites and is specifically enriched for known or putative xenometabolites; thus, the platform has been termed the XenoScan (Mercer et al., 2020). Further identification of metabolites was conducted with mzCloud and mzVault spectral libraries. Structurally identified metabolites were given the following ranking according to the standard published previously (Blazenovic et al., 2018; Schymanski et al., 2014): (1) accurate mass, retention time, and MS^2^ spectra matched to in‐house standard, (2) accurate mass and MS^2^ spectra matched to known standard (internal or cloud) without retention time matched. Metabolites that did not meet rankings 1 and 2 were removed from analysis, resulting in 189 and 103 total metabolites identified in stool and serum samples, respectively.

### 2.6 Statistics

Relative standard deviations (RSD) were calculated using QC replicates and metabolites with >30% RSD (Peterson, 2016) in the QC pool replicates were filtered from the analysis. This resulted in the removal of 10 and 12 metabolites in serum and stool, respectively. The effect of exercise training on the fecal and serum metabolome was assessed using a 10‐fold cross validated partial least squares discriminant analysis (PLS‐DA) with one latent variable using residuals from a linear mixed model to adjust for only BMI classification. Data were split into training and test (75/25% split), where model fit and feature selection was done entirely in the training data. Features were selected if their variable importance in projection (VIP) score was greater than 1 (Chong, 2005). These feature‐selected metabolites were then used to fit a reduced PLS‐DA model. Overall percent accuracies with 95% confidence intervals were used to assess model performance and indications of overfitting using the validation data. Overfitting was considered if 95% confidence intervals percent accuracy of 60% or less. Each metabolite was analyzed by linear mixed models (LMMs) to assess the effect of the exercise intervention. LMMs included exercise intervention (pre/post), BMI classification (obese/lean), sex (M/F), and study cohort (1/2), and the exercise × BMI interaction as coefficients; subject ID was included as the random effect. Correction for multiple comparisons was applied using Benjamini and Hochberg's false discovery rate (FDR) correction. Statistical modeling of metabolomics data was conducted in the R Statistical Language (v4.1.0). To further unravel broader patterns between metabolites and their responsiveness to exercise, hierarchical cluster analysis (HCA) was implemented as previously described using Metaboanalyst (Metaboanalyst.com) software (Morville et al., 2020). Data were normalized (log transformation, autoscaled [mean‐centered and divided by the standard deviation of each variable]) and similarity was calculated with Euclidean distances, with Ward's minimum variance method. Hierarchical clustering was performed with the *hclust* function in package *stat* using fuzzy c‐means algorithm with the optimal parameters with *c* = 6 and *m* = 3 in Metaboanalyst. Metabolites within each cluster were then analyzed by KEGG metabolic pathway analysis to determine patterns of metabolic shifts in response to exercise in lean and obese participants.

## 3 RESULTS

### 3.1 Exercise training modifies fecal metabolome with response partially dependent on obesity status

**FIGURE 1:**
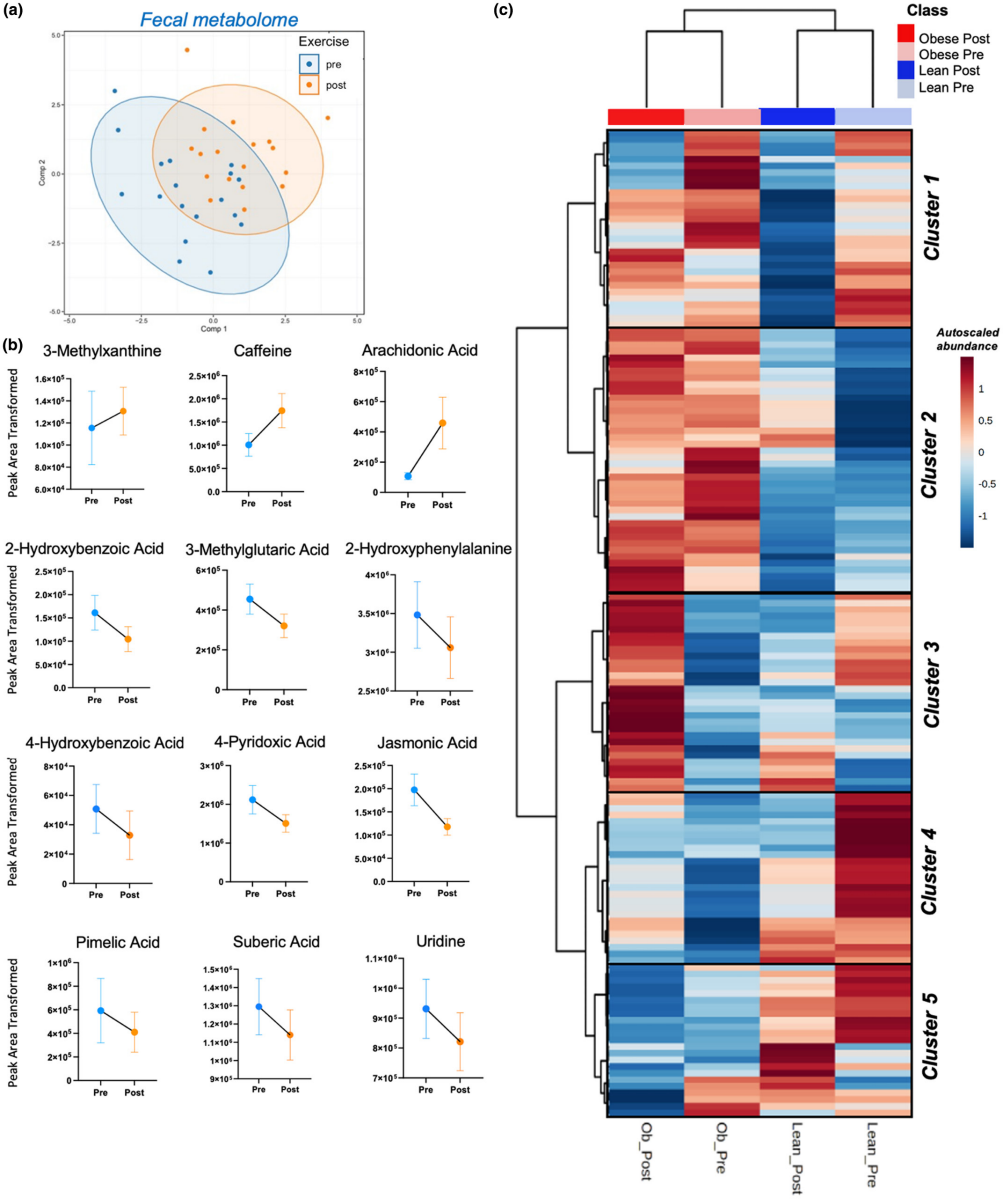
Fecal metabolome responses to exercise training. (a). Partial least squares discriminant analysis (PLS‐DA) was used to assess metabolic profiles of feces collected before—(Pre‐blue) and after—(Post‐orange) a 6‐week aerobic exercise intervention. Residuals from linear mixed models accounting for obesity status, sex, and study cohort were used in PLS‐DA models. Axes indicate values for PLS‐DA scores (i.e., sample projections). Metabolites were selected for inclusion if Variable Importance in Projection (VIP) value was > 1. All model development, feature selection, and visualizations were conducted on training data (*n* = 18). (b) Linear mixed models (LMM) identified fecal metabolites responsive to exercise training (regardless of obesity, sex, age or study cohort). (c) Cluster analysis revealed 5 primary metabolite subgroups that shifted concordantly as a result of exercise training.

We first implemented PLS‐DA to discern whether metabolite shifts occurred in response to the exercise intervention. Inclusion of all high‐confidence annotated metabolites (146) in the model, where intensity was determined by relative peak area, resulted in a good average predicted accuracy of held‐out samples (83%). However, because the prediction accuracy confidence interval indicated insufficient model convergence (95% CI: 52%, 98%) on the validation data set, we introduced a reduced model with 16 fecal metabolites with VIP values >1, resulting in an improved confidence interval of predicted accuracy (CI: 74%, 100%; Figure 1a).

Linear mixed models (LMMs) were then implemented with adjustments for study cohort, obesity status (lean or obese), and sex (male or female) to determine whether the effect of the exercise intervention on the fecal metabolome would remain apparent when accounting for these factors. In total, LMMs identified 12 fecal metabolites that were responsive to exercise training regardless of BMI (main effect exercise FDR <0.05; Figure 1b): 3‐methylxanthine, caffeine, and arachidonic acid increased following the intervention, whereas 2‐hydroxybenzoic acid, 3‐methylglutaric acid, 2‐hydroxyphenylalanine, 4‐hydroxybenzoic acid, 4‐pyridoxic acid, jasmonic acid, pimelic acid, suberic acid, and uridine decreased in response to the intervention (Figure 1b).

**FIGURE 2:**
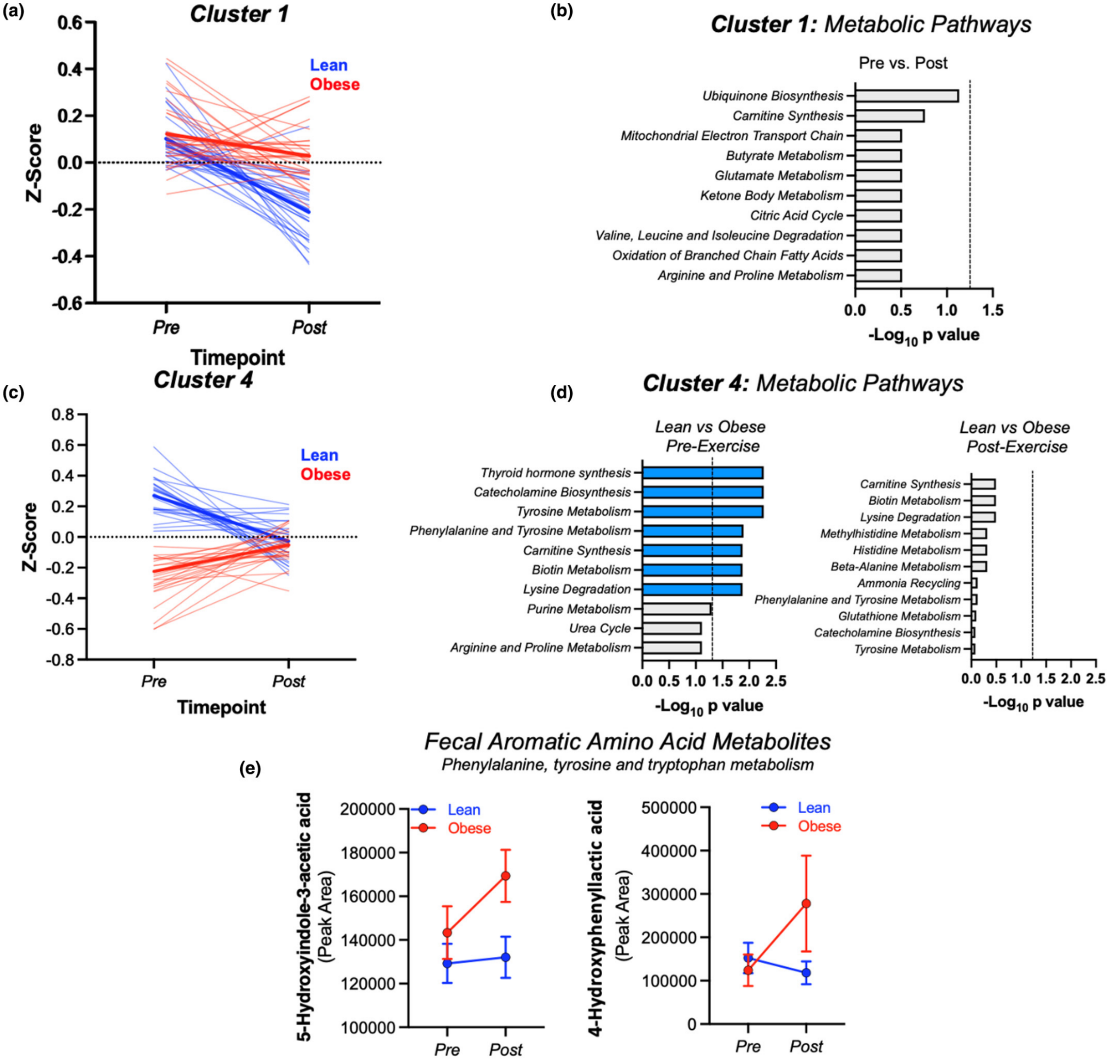
Fecal metabolic pathway responses to exercise training. (a). *Z*‐scores of Cluster 1 reveals metabolites that decreased in feces as a result of exercise training. (b) No KEGG pathways were significantly modified by exercise within cluster 1. (c). *Z*‐score of Cluster 4 reveals a group of metabolites that responded differentially to exercise training based on obesity status. (d) KEGG pathway analysis reveals pathways within cluster 4 that differed by obesity status pre‐exercise (left, blue bars represent significant obesity effect at ‐log10 *p*‐value >1.25) but were no longer different post‐exercise (right). (e) Fecal metabolites within aromatic amino acid (ArAA) pathways responded differentially to exercise training based on obesity status (LMM obesity × exercise effect *p* < 0.05).

Next, we aimed to understand whether obesity impacted functional metabolic responses to exercise training. To fully understand functional shifts in fecal metabolome in response to exercise, we implemented two‐way hierarchical cluster analysis (HCA) merged with KEGG metabolic pathway analysis. HCA allowed for stratification of metabolites grouped by pre‐ and post‐exercise conditions within lean and obese individuals. This analysis identified five main groups (clusters) of metabolites that responded to exercise training differently in lean and obese participants (Figure 1c). *Z*‐scores were calculated for all metabolite clusters to characterize metabolite responses to exercise followed by KEGG pathway analysis to determine the effects of exercise and obesity within each cluster (e.g., *Clusters 1 and 4*, Figure 2a‐e; *Clusters 2*, *3*, and *5*, Figure S1).

Examination of *Cluster 1* revealed a group of fecal metabolites that largely decreased in response to exercise regardless of obesity status (Figures 1c and 2a). Pathway analysis indicated *ubiquinone biosynthesis* and *carnitine synthesis* as the most affected by exercise training, although these did not reach statistical significance following FDR correction (Figure 2b).

*Cluster 4* contained a group of metabolites that responded differentially to exercise based on obesity status, generally increased in obese individuals but decreased in participants without obesity (Figures 1c and 2c). Metabolic pathway analysis of fecal metabolites prior to exercise training revealed significant differences, based on obesity status, in pathways related to *thyroid hormone biosynthesis*, *catecholamine production*, and *aromatic amino acid* (ArAA) metabolism, among others (Figure 2d; left insert). However, after exercise training, these pathway differences between lean and obese participants were abolished (Figure 2d; right insert), indicating a differential metabolic response to exercise training based on obesity status. Within *Cluster 4* we identified a serotonin metabolite, 5‐hyroxyindole‐3‐acetic acid (5‐HIAA), and a microbial tyrosine metabolite, 4‐hydroxyphenyllactic acid (4‐HPLA), which responded differentially to exercise based on obesity status (Figure 2e). *Clusters 2*, *3*, and *5* also contained metabolite groups that were responsive to exercise training, but KEGG analysis did not reveal any coordinated shifts in metabolic pathways (Figure S1).

### 3.2 Exercise training‐induced modification to overnight‐fasted serum metabolome indicates changes in microbial amino acid metabolism

**FIGURE 3:**
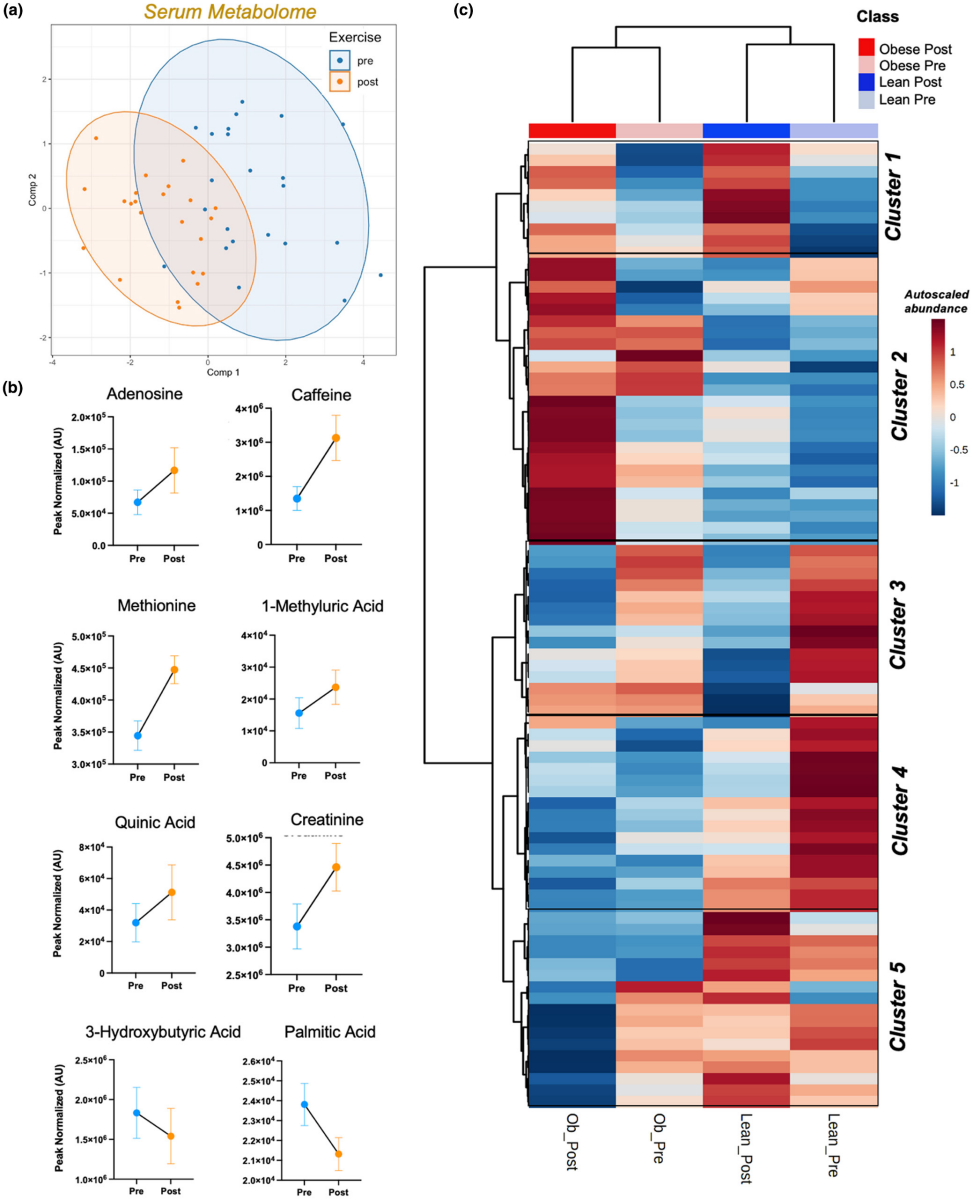
Serum metabolome responses to exercise training. (a). Partial least squares discriminant analysis (PLS‐DA) was used to assess metabolic profiles of feces collected before—(Pre‐blue) and after—(Post‐orange) a 6‐week aerobic exercise intervention. Residuals from linear mixed models accounting for obesity status, sex, and study cohort were used in PLS‐DA models. Axes indicate values for PLS‐DA scores (i.e., sample projections). Metabolites were selected for inclusion if Variable Importance in Projection (VIP) value was >1. All model development, feature selection, and visualizations were conducted on training data (*n* = 18). (b) Linear mixed models (LMM) identified serum metabolites responsive to exercise (regardless of obesity, sex, age or study cohort). (c) Cluster analysis revealed serum metabolite subgroups that shifted concordantly as a result of exercise training.

We next used PLS‐DA to analyze serum metabolic responses to exercise training. This model resulted in a good overall accuracy average of held‐out data (90%), but also had a wide confidence interval (CI: 56%, 100%). Thus, metabolites with VIP loadings >1 were utilized to refine the model (Figure 3a); however, the reduced model still had a wide confidence interval (CI: 36%, 100%). We implemented LMMs, which identified eight serum metabolites with a significant main effect of exercise (*p* < 0.05). Adenosine, caffeine, methionine, 1‐methyluric acid, quinic acid, and creatinine increased after the intervention and 3‐hydroxybutyric acid and palmitic acid decreased (Figure 3b). These patterns occurred regardless of obesity status and thus are attributed to the effect of exercise intervention specifically.

**FIGURE 4:**
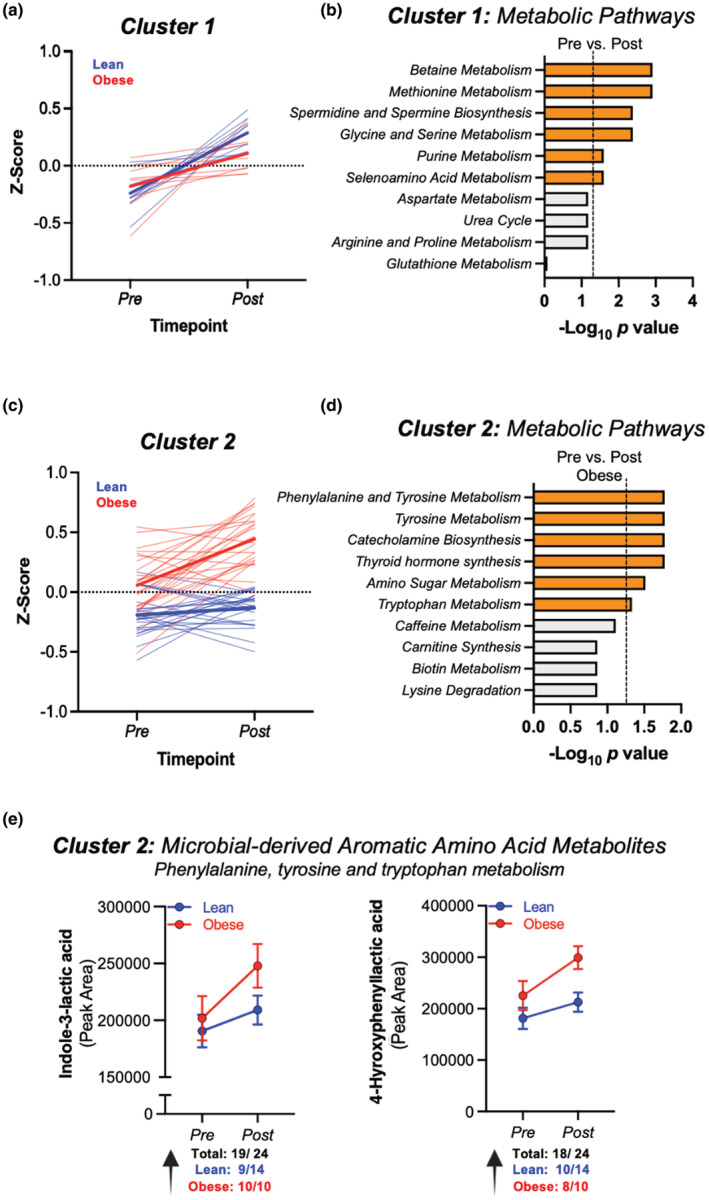
Serum metabolic pathway responses to exercise training. (a). *Z*‐scores of Cluster 1 reveals a group of metabolites that responded to exercise training regardless of obesity status. (b) KEGG pathway analysis reveals pathways within cluster 1 that differed pre to post‐exercise training. (c). Analysis of Cluster 2 revealed a group of metabolites responsive to exercise training that was partially dependent on obesity status. (d) KEGG pathway analysis reveals metabolic pathways within Cluster 2 that shifted in response to exercise training. (e) Microbial‐derived aromatic amino acid (ArAA) metabolites, indole‐3‐lactic acid and 4‐hydroxyphenyllactic acid (4‐HPLA) were increased by exercise in the serum of both lean and obese participants (main effect Exercise *p* < 0.05). Right inserts represent percent (%) change from pre–post‐exercise in lean and obese participants.

To interrogate functional shifts in the serum metabolome in response to exercise, and the potential impact of obesity on these patterns, we similarly implemented two‐way HCA merged with KEGG metabolic pathway analysis and performed *Z*‐score calculations within each cluster to determine whether groups of metabolites responded in parallel to exercise (Figure 3, *Clusters 1–2*, Figure 4a–e; *Clusters 3–5*, Figure S2). *Cluster 1* analysis revealed significant increases by exercise—regardless of obesity status—on betaine, methionine, spermine, glycine/serine, purine, and selenoamino acid metabolic pathways (Figure 4a, b). *Cluster 2* analysis revealed a group of metabolites that increased in response to exercise training, primarily in obese versus lean participants (Figure 4c). Pathway analysis of *Cluster 2* indicated that metabolites associated with *aromatic and amino acid metabolism* and *catecholamine biosynthesis*, among others, were significantly upregulated in response to exercise training in obese participants (Figure 4d). Next, we focused on known microbial ArAA metabolites in the serum and found two microbially derived metabolites downstream of the ArAAs tyrosine and tryptophan—4‐hydroxyphenyllactic acid (HPLA) and indole‐3‐lactic acid (ILA), respectively—which were significantly increased by exercise training (Figure 4e; ANOVA main effect exercise FDR *p* < 0.05). ILA and 4‐HPLA increased more substantially in obese versus lean participants; however, this interaction did not reach statistical significance (exercise × obesity *p* > 0.05). Analysis of *Clusters 3–5* revealed shifts of serum metabolites in response to exercise and in relation to obesity status, but further KEGG mapping did not reveal any significant changes in functional metabolic pathways within these clusters (Figure S2).

### 3.3 Fecal and serum aromatic amino acid metabolites associate with body composition, markers of insulin sensitivity, and cardiorespiratory fitness

**FIGURE 5:**
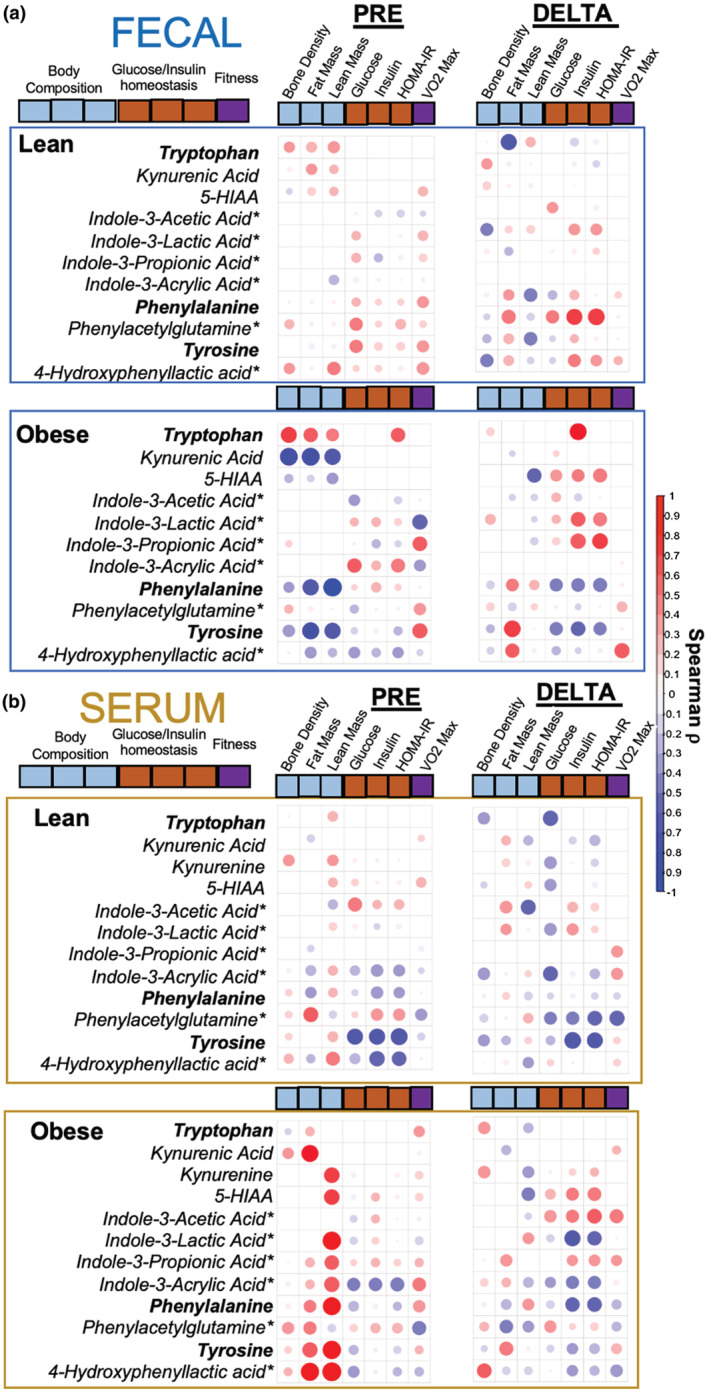
Associations between microbial‐derived aromatic amino acid (ArAA) metabolites and physiological outcomes. Colorimetric scaling of significant Spearman mo (*ρ*) correlation coefficients relating ArAA and microbial‐derived metabolites (*) to body composition (bone density, fat mass, lean mass), glucose homeostasis (fasting glucose, fasting insulin and HOMA‐IR) and fitness outcome variables ($\dot{V}O_{2\max}$) at baseline (Pre) and in response (Delta) to 6 weeks of exercise training in (a) fecal samples and (b) serum samples. Delta correlations are Δ_Post‐Pre metabolite_ vs. Δ_Post‐Pre phenotype_. Dot size and color represent Spearman Δ coefficient (^−^1 Large bright blue to +1 Large bright red). Squares containing dots represent significance at *p* < 0.05. Blank squares *p* > 0.05. *Microbial‐derived/modified.

ArAA‐derived metabolites were responsive to exercise training in both feces and serum. Since exercise typically results in reduced body fat, improved insulin sensitivity, and increased levels of fitness, we next explored whether these ArAA metabolites correlated with body composition (bone density, fat mass, lean mass), markers of fasting glucose homeostasis (glucose, insulin, HOMA‐IR), and cardiorespiratory fitness ($\dot{V}O_{2\max}$) across all participants. We measured relationships of ArAA and microbial derivatives to phenotypic outcomes before exercise training (Pre_metabolite_ vs. Pre_phenotype_; left panel), and in response to exercise training (Δ _Post‐Pre metabolite_ vs. Δ _Post‐Pre phenotype;_ right panel) in both lean and obese participants (Figure 5a,b).

Globally, fecal ArAAs and downstream xenometabolites associated with phenotypic outcomes at baseline and in response to exercise (Figure 5a). For example, fecal indole‐3‐propionic acid positively associated with $\dot{V}O_{2\max}$ at baseline regardless of obesity status (Spearman *⍴* = 0.38, *p* < 0.05). However, further analysis revealed relationships between fecal ArAAs and phenotypic outcomes that were largely dependent on BMI status. For example, exercise‐induced changes in phenylacetylglutamine positively associated with changes to HOMA‐IR in lean (Spearman *⍴* = 0.833, *p* < 0.01), but not obese (Spearman *⍴* = 0.32, *p* > 0.05), participants.

In the serum, correlational analysis revealed a pattern whereby many ArAAs and downstream metabolites associated with host phenotype (Figure 5b). For example, many microbially derived ArAA metabolites positively associated with lean mass at pre‐exercise timepoints, regardless of obesity status. This includes exercise‐responsive metabolites ILA and 4‐HPLA, which were strongly associated with lean mass at baseline when collapsed across both BMI participant groups (Spearman ⍴, Lean mass vs. ILA = 0.46, 4‐HPLA = 0.70, *p* < 0.05; Figure 5b; left panels). Changes (Δ_Post‐Pre_ exercise) in serum ArAAs and metabolites also paralleled changes to phenotypic outcomes in response to exercise training (Figure 5b; right panels). However, these relationships were somewhat divergent in lean versus obese participants. For instance, exercise‐induced increases in ILA and 4‐HPLA strongly paralleled reductions in fasting insulin in obese participants (Spearman ⍴; Insulin Δ vs. ILA Δ = −0.71, 4‐HPLA Δ = −0.49, *p* < 0.05) but not in lean participants (*p* > 0.05; Figure 5b).

**FIGURE 6:**
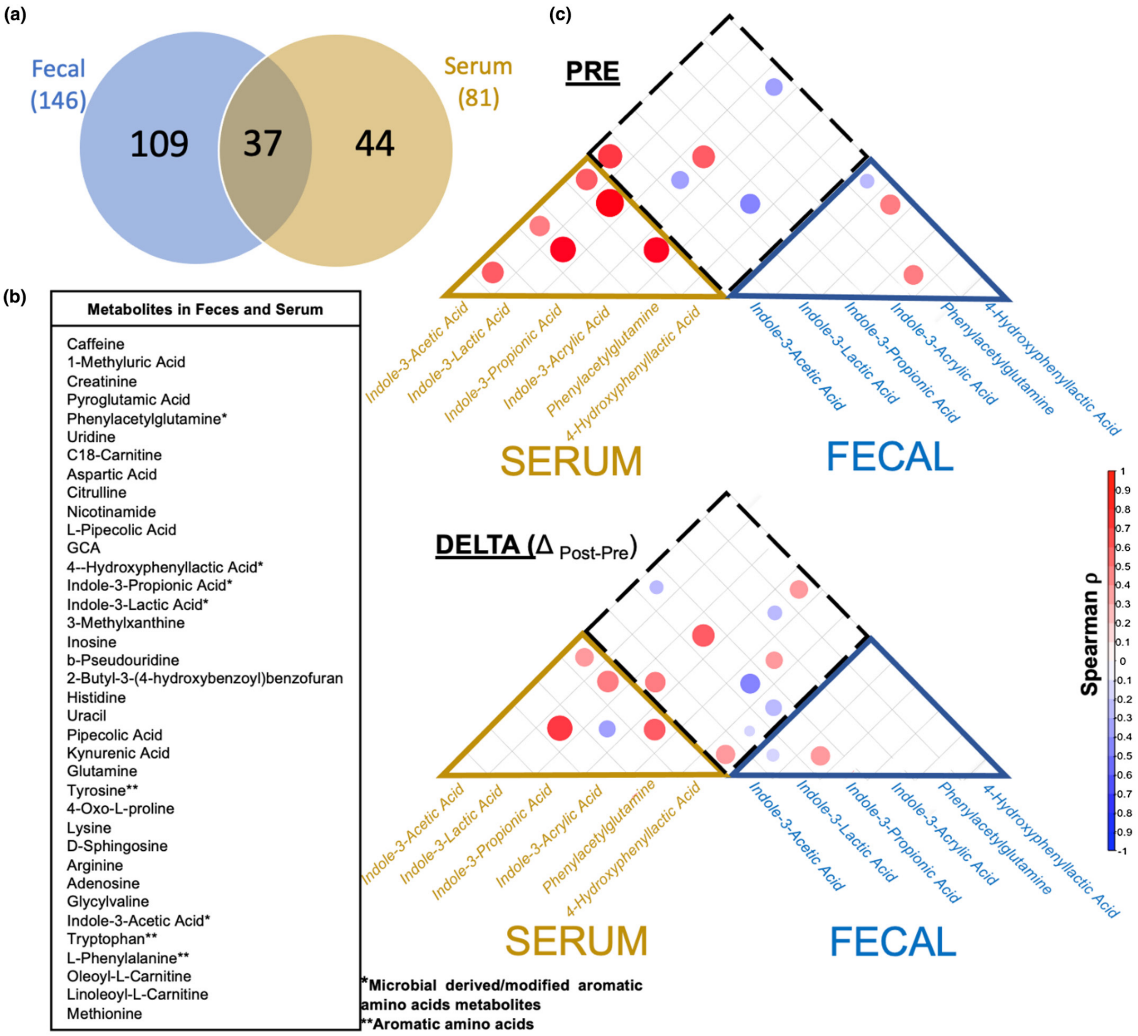
Metabolites identified in both feces and serum, and fecal‐serum correlations for aromatic amino acid (ArAA) metabolites. (a) Venn Diagram displaying the number of metabolites found in feces, serum, and both feces and serum samples. (b) Complete list of the 37 metabolites found in both fecal and serum samples. (c) Colorimetric scaling of significant Spearman rho (*ρ*) correlation coefficients relating microbial‐derived metabolites to inter and intra tissue (serum and feces) at baseline (Pre) and in response (Delta) to 6 weeks of exercise training. Dot size and color represent Spearman Δ coefficient (^−^1 Large bright blue to +1 Large bright red). Squares containing dots represent significance at *p* < 0.05. Blank squares *p* > 0.05.

Regardless of participant BMI status, ArAAs and ArAA metabolites were found in both fecal and serum samples. A total of 37 metabolites were found at both sites, including tyrosine, tryptophan, and phenylalanine, as well as 5 microbe‐derived ArAA metabolites (Figure 6a,b). Metabolite correlation analysis, collapsed across both BMI groups, revealed associations between ArAA metabolites at baseline and in response to exercise (Figure 6c). For example, in the serum, concentrations of 4‐HPLA and ILA (Pre and Δ post‐pre) were highly correlated, further suggesting shared metabolic regulation of these two compounds (*p* < 0.01; Figure 6c). To our surprise, however, inter‐site analysis (serum vs. fecal) of ArAA metabolites revealed fewer associations. For example, fecal concentrations of ILA and 4‐HPLA were not significantly correlated with serum concentrations of these same metabolites (Figure 6c). Accordingly, fecal and serum metabolite concentrations also had disparate correlations to body composition. For example, within obese subjects, fecal and serum ArAA metabolites showed opposite trends in relation to body composition.

## 4 DISCUSSION

Exercise training improves fitness while promoting metabolic and immune health, but the molecular and signaling mechanisms are not fully elucidated. These benefits may be mediated, at least in part, through exercise training‐associated adaptations in circulating and tissue‐specific metabolites. Herein, we provide evidence that microbial‐ and host‐derived metabolites found in both feces and serum respond to aerobic exercise training in previously sedentary adults. We also discovered that obesity status is an important factor that contributes to specific serum and fecal metabolite responses to exercise training.

Exercise training modified several fecal metabolites regardless of obesity, age, or sex. Metabolite cluster and pathway analysis of the fecal metabolome indicated that ubiquinone pathways were most affected by exercise. This included a significant reduction in 4‐hydroxybenzoic acid (4‐HB) by exercise training regardless of obesity status. Of note, 4‐HB has been suggested to serve as a precursor to the benzoquinone ring of Coenzyme Q in animals, yeast, and bacteria (Parson & Rudney, 1964). Because 4‐HB is a cross‐species metabolite, these changes may have relevance for bacterial electron transport and/or mitochondrial turnover in the local gut environment, among other possibilities. The origin of 4‐HB (host vs. microbe) is still currently unknown.

Additional fecal metabolites were altered by exercise training. Among them, 3‐methylglutaric (3‐MG) acid is a downstream product of leucine metabolism and was significantly decreased with exercise training. 3‐MG acid accumulation induces reactive oxygen species production, ultimately leading to mitochondrial dysfunction and tissue damage (Colin‐Gonzalez et al., 2016; Jones et al., 2020; Ribeiro et al., 2011). However, the physiological relevance of 3‐MG acid is not well studied, especially in context of the microbiome, and thus should be examined more closely in future studies. We also found that caffeine and downstream metabolites (e.g. 3‐methylxanthine, quinic and methyluric acid) were increased in both the feces and serum in response to exercise training. While caffeine itself is not a microbial metabolite, other xanthine metabolites, including those upregulated in this study with exercise training, are known to be modified by microbial species (Zhou et al., 2020). Recent data indicates that changes in microbial xanthine metabolism is a key feature of select chronic states including chronic fatigue syndrome, where disease progression decreases microbial xanthine metabolism (Xiong et al., 2023). Conversely, the current study provides evidence that microbial xanthine metabolite production is increased in response to exercise training. These data coincide with our previous report showing caffeic acid metabolites were upregulated in response to acute exercise without any changes to dietary caffeine intake (Grapov et al., 2019).While the mechanisms leading to exercise‐induced shifts in xanthine metabolism remain unclear, microbial xanthine metabolism is a potential regulator of host physiology and should be examined more closely in future studies.

In accordance with our previous work showing differential effects of exercise on gut microbiome composition when comparing lean and obese individuals (Allen et al., 1985), obesity status appeared to affect fecal metabolite profiles in response to exercise. Cluster and pathway analysis revealed that these effects were driven primarily by metabolites within catecholamine biosynthetic and ArAA metabolic pathways. Within ArAA metabolism, 5‐hyroxyindoleacetic acid (5‐HIAA) is a downstream product of serotonin and was significantly increased in obese (but not lean) participants in response to exercise training. 4‐hydroxyphenyllatic acid (4‐HPLA) is a microbial metabolite of tyrosine and showed similar trends to 5‐HIAA in response to exercise. The physiological relevance of these findings is in need of future investigation, but the data herein illustrate that exercise effects on microbiome metabolism can be influenced by obesity and related sequela.

The serum metabolome was also responsive to exercise training with some effects dependent on obesity status. Cluster analysis revealed many pathways responsive to exercise regardless of obesity status. These included metabolites involved in purine synthesis pathways (1‐methyluric acid, 3‐methylxanthine). Other studies have indicated an increase in purine metabolites and changes to metabolic pathways associated with purine metabolism in response to both acute exercise and exercise training (Dudzinska, Lubkowska, Dolegowska, & Safranow, 2010; Dudzinska, Lubkowska, Dolegowska, Safranow, & Jakubowska, 2010; Pospieszna et al., 2019). The mechanisms involved are unclear, but we speculate that the adenosine monophosphate (AMP) catabolic pathway may be upregulated with exercise training in various tissues, including the gut.

Aromatic amino acid metabolites with known microbial origin, indole‐3‐lactic acid (ILA) and 4‐hydroxyphenyllactic acid (4‐HPLA), were upregulated in the serum of exercise‐trained individuals versus pre‐training levels. Both ILA and 4‐HPLA are downstream metabolites of tryptophan and tyrosine, respectively, and their production relies on similar enzymatic machinery in the gut microbiome. Phenyllactate dehydrogenase (fLDH) is an enzyme present in select lactic acid‐producing bacteria (LAB), including some species of *Bifidobacterium* and *Lactobacillus*, and contributes to the production of ILA and 4‐HPLA from ArAA precursors (Dodd et al., 2017; Sakurai et al., 2021). We have not previously observed any changes to these two LAB genera in response to exercise in human feces (Allen et al., 2018). However, *Bifidobacterium* and *Lactobacillus* species are found in highest concentrations in the ileum and thus fecal analysis may not adequately represent exercise‐induced shifts to the upper gastrointestinal microbiome. Here, we speculate that exercise‐induced increases in serum ILA and 4‐HPLA may result from an increased abundance of *Bifidobacterium*, *Lactobacillus*, and/or increased fLDH enzyme activity within the ileum.

In support of this latter hypothesis, we have also previously reported increases in circulating ILA during recovery from an acute exercise bout (Grapov et al., 2019). However, the mechanisms underlying increases in serum ILA and 4‐HPLA by acute exercise and/or exercise training are currently unknown and need further investigation. Notably, we observed no correlation between fecal and serum levels of ILA and 4‐HPLA in the present study. As the fecal concentration of many gut metabolites depends on gut transit time, cross‐feeding interactions between microbes, and the rate of host absorption (Donia & Fischbach, 2015), sampling multiple regions along the GI tract in addition to serum across multiple longitudinal timepoints may be required to fully understand the impacts of these metabolites on host health.

Regardless of the mechanisms underlying these responses, ILA and 4‐HPLA both exhibit immunomodulatory and metabolic tuning properties and thus signify physiologically relevant shifts in microbial metabolism induced by exercise training. For instance, ILA has been shown to regulate inflammation by scavenging free radicals and inhibiting the production of interleukin‐6, a pro‐inflammatory cytokine (Aoki‐Yoshida et al., 2013; Meng et al., 2020). In addition, 4‐HPLA has been shown to attenuate reactive oxygen species production in neutrophils (Beloborodova et al., 2012). Future studies are needed to confirm such changes and explore the relationship between microbially derived ArAA metabolites and health outcomes.

In conclusion, we found that 6 weeks of aerobic exercise training shifts both fecal and serum metabolome in previously sedentary adults, despite no discernable changes in dietary patterns within individual participants or microbial community structure. Exercise‐induced changes in metabolomic profiles were partially dependent on baseline BMI status. Metabolites associated with ArAA metabolism were most responsive to exercise in both feces and serum. These included bioactive metabolites with known microbial origin and potential health‐modifying effects. These data highlight the importance of understanding the microbiome and xenometabolome when investigating responses to exercise training. Future studies are needed to further understand the potential role of microbial‐derived metabolites in mediating exercise adaptations and physiological pathways relevant to human health. Such experiments could explore the therapeutic potential of these metabolites as “postbiotics” (Swanson et al., 2020). The specific communication pathways and host‐derived signals that drive the associations remain to be determined.

### 4.1 Limitations

We acknowledge the limitations of this study. This study represents a relatively small sample size with only 24 participants (*n* = 15 lean and *n* = 9 obese). While participants followed 3‐day diet controls prior to all sample collections and were instructed to maintain overall dietary patterns including caffeinated beverage consumption, we cannot completely rule out variation in diet control. Furthermore, due to the heterogeneity of baseline metabolome profiles, results from these studies should be interpreted with some caution and merit follow‐up analysis with larger study populations. We also acknowledge that untargeted metabolomic analysis should be followed up with more targeted approaches in future studies. Finally, since we did not collect feces quantitatively (i.e., total dry weight per a given time or total sample), we could not calculate the total pool size or daily excretion for each metabolite. Nevertheless, the novel patterns observed here are reflective of diet‐independent exercise intervention effects on xenometabolism and the impact of obesity status on these changes.

## AUTHOR CONTRIBUTIONS

Mikaela C. Kasperek, Lucy Mailing, Brian D. Piccolo, Jeffery A.Woods, Sean H. Adams, and Jacob M. Allen were responsible for conception and design of the work. Mikaela C. Kasperek, Lucy Mailing, Brian D. Piccolo, Becky Moody, Renny Lan, Lucy Mailing, Xiaotian Gao, Diego Hernandez‐Saavedra, Jeffrey A. Woods, Sean H. Adams, and Jacob M. Allen were responsible for acquisition, analysis or interpretation of data for the work. Mikaela C. Kasperek, Lucy Mailing, Brian D. Piccolo, Becky Moody, Renny Lan, Lucy Mailing, Xiaotian Gao, Diego Hernandez‐Saavedra, Jeffrey A. Woods, Sean H. Adams, and Jacob M. Allen were responsible for drafting the work or revising it critically for important intellectual content. Mikaela C. Kasperek, Lucy Mailing, Brian D. Piccolo, Becky Moody, Renny Lan, Xiaotian Gao, Diego Hernandez‐Saavedra, Jeffrey A. Woods, Sean H. Adams, and Jacob M. Allen were responsible for drafting the work or revising it critically for important intellectual content. All authors approved the final version of the manuscript; agreed to be accountable for all aspects of the work in ensuring that questions related to the accuracy or integrity of any part of the work are appropriately investigated and resolved; and all persons designated as authors qualify for authorship, and all those who qualify for authorship are listed.

## CONFLICT OF INTEREST STATEMENT

S.H. Adams is founder and principal of XenoMed, LLC, which is focused on research and discovery in the area of microbial metabolism. XenoMed had no part in the research design, funding, results or writing of the manuscript.

## ETHICS STATEMENT

This study was approved by the University of Illinois Urbana‐Champaign Institutional Review Board, written informed consent was obtained from all participants, and all procedures and protocols conformed to the standards of use of human participants in research as outlined in the Sixth Declaration of Helsinki.

## Supporting information


**Figure S1** (a–c) Hierarchical Cluster analysis (HCA) of fecal metabolite subgroups that clustered according to obesity status or shifted concordantly as a result of exercise training. (d–f) KEGG pathway analysis of each fecal metabolite cluster. (g) Complete list of fecal metabolite clusters 1–5.
**Figure S2**.(a–c) Cluster analysis of serum metabolite subgroups that clustered according to obesity status or shifted concordantly as a result of exercise training. (d–f) KEGG pathway analysis of each serum metabolite cluster. (g) Complete list of serum metabolite clusters 1–5.
