# Supplementary figures and images for: Exercise training modifies xenometabolites in gut and circulation of lean and obese adults
Source: Physiol Rep. 2023 Mar 22;11(6):e15638. doi: 10.14814/phy2.15638 (PMC10031301; doi:10.14814/phy2.15638)

**
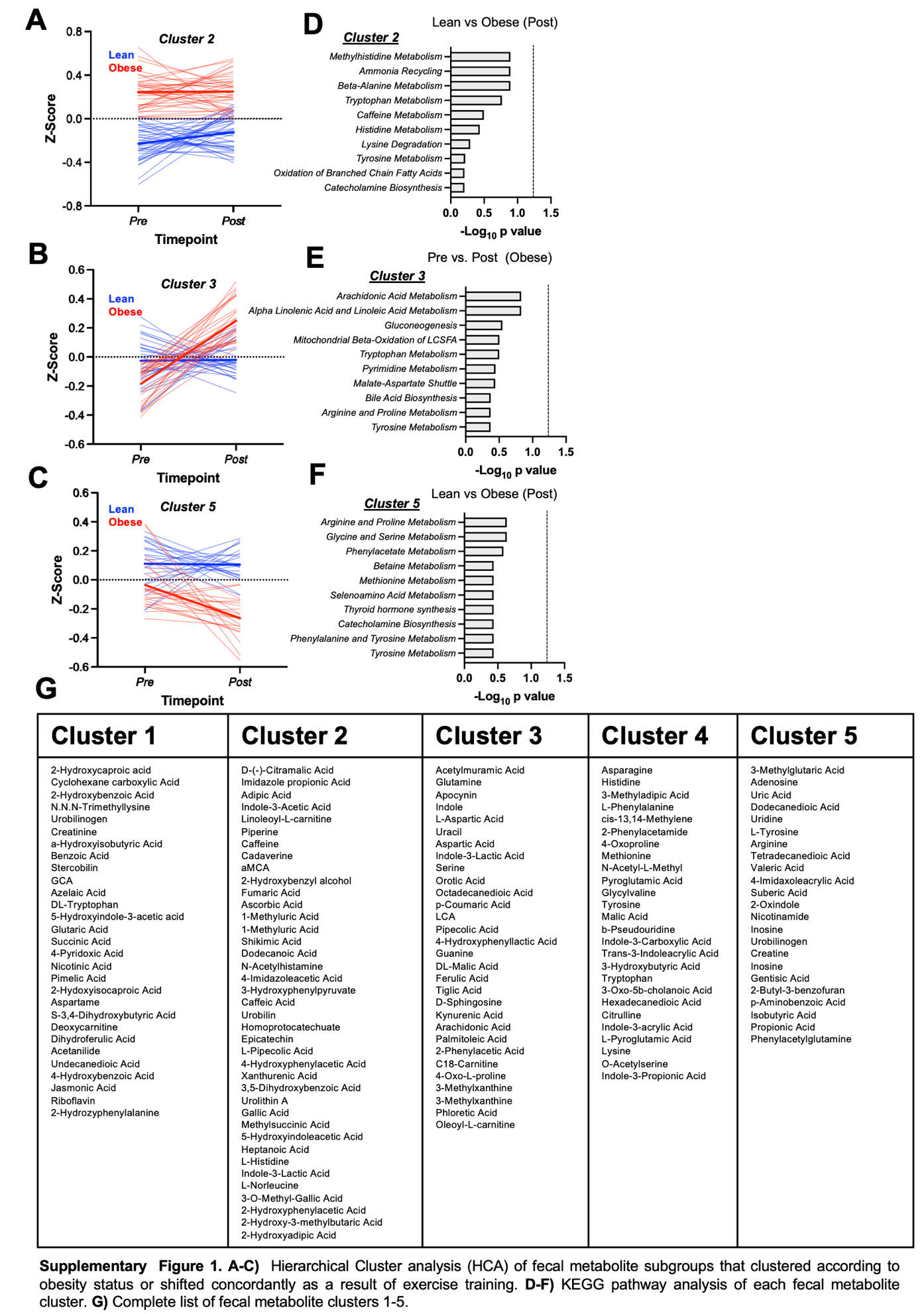
**

**
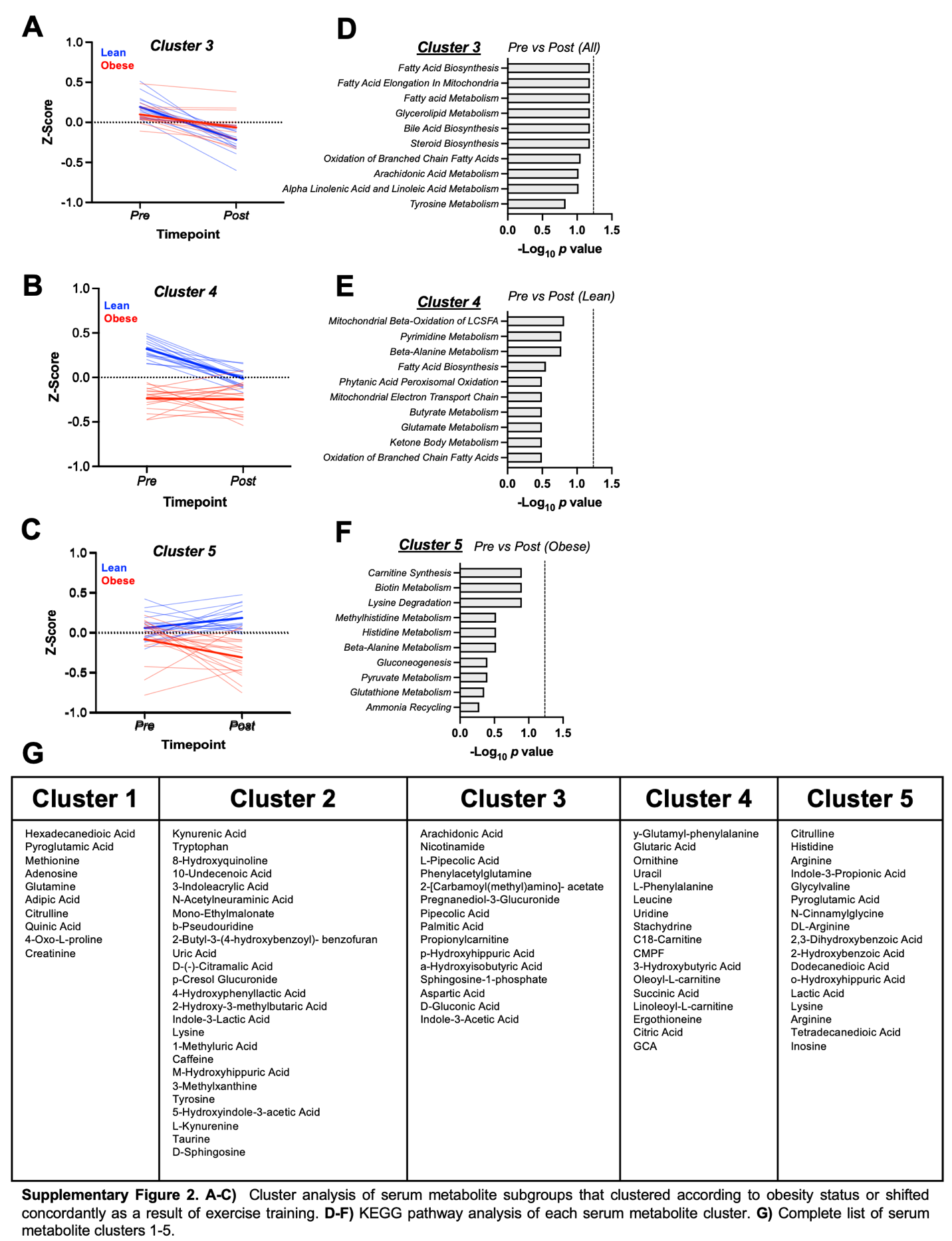
**

Supplement: Supplementary file 1 — Figure S1 (a–c) Hierarchical Cluster analysis (HCA) of fecal metabolite subgroups that clustered according to obesity status or shifted concordantly as a result of exercise training. (d–f) KEGG pathway analysis of each fecal metabolite cluster. (g) Complete list of fecal metabolite clusters 1–5. Figure S2.(a–c) Cluster analysis of serum metabolite subgroups that clustered according to obesity status or shifted concordantly as a result of exercise training. (d–f) KEGG pathway analysis of each serum metabolite cluster. (g) Complete list of serum metabolite clusters 1–5. [file PHY2-11-e15638-s001.docx]
